# Characteristics and Incidence of Concussion Among a US Collegiate Undergraduate Population

**DOI:** 10.1001/jamanetworkopen.2019.17626

**Published:** 2019-12-18

**Authors:** John Breck, Adam Bohr, Sourav Poddar, Matthew B. McQueen, Tracy Casault

**Affiliations:** 1 Medical Services at University of Colorado, Boulder,; 2 Department of Integrative Physiology, University of Colorado, Boulder; 3 Department of Family Medicine and Orthopedics, University of Colorado, Denver

## Abstract

**Importance:**

Concussion on university campuses is a significant health problem. Characterizing the incidence of concussion on college campuses may inform education and resource allocation policy at student health care centers.

**Objectives:**

To establish a measure of concussion incidence among collegiate undergraduate students and to describe characteristics associated with concussion incidence, including sex, cause, and month.

**Design, Setting, and Participants:**

This prospective cohort study included data from 3 academic years from August 2015 to April 2018 at a large, public university in the United States. Participants included any undergraduate student or varsity athlete who was diagnosed with at least 1 concussion during the academic year.

**Exposures:**

Sport- and non–sport-related activities of undergraduate students.

**Main Outcomes and Measures:**

Concussion diagnosis.

**Results:**

Among 954 undergraduate students from the general undergraduate population with at least 1 concussion, including 502 men and 452 women, 1020 concussions were diagnosed in 3 academic years. During 2 academic years, a total of 80 concussions occurred among the varsity athlete population, including 26 men and 54 women. Overall, concussion incidence among both the general undergraduate population and varsity athletes was 132.4 (95% CI, 123.2-142.0) concussions per 10 000 students. Men sustained concussions at a rate of 126.1 (95% CI, 114.1-139.0) concussions per 10 000 students and women sustained concussions at a rate of 140.0 (95% CI, 126.2-155.3) concussions per 10 000 students for the 2016 to 2017 and 2017 to 2018 academic years. Concussion incidence peaked in August at the start of the academic year and the rate of non–sport-related concussions (81.0 [95% CI, 73.9-88.7] concussions per 10 000 students for academic years 2016-2017 and 2017-2018) was higher than the rate of sport-related concussions (51.5 [95% CI, 49.5-57.7] concussions per 10 000 students for academic years 2016-2017 and 2017-2018).

**Conclusions and Relevance:**

This cohort study found concussions to be common among this US collegiate population. While concussion is often associated with sport, the incidence of non–sport-related concussion was higher than that of sport-related concussion throughout the academic year. Additional research is warranted to determine if this incidence measure among undergraduate students is generalizable to other university populations.

## Key Points

**Question:**

What is the incidence of concussion among undergraduate students at a large, public university in the United States?

**Findings:**

In this cohort study of 954 undergraduate college students and 80 varsity athletes diagnosed with at least 1 concussion during the academic year, incidence was found to be 132.4 concussions per 10 000 students. For the general student population, incidence was highest in August, and overall, the rate of sport-related concussion was lower than the rate of non–sport-related concussion.

**Meaning:**

Concussion incidence at a public university is common and higher than reported rates in the general population.

## Teaser

This cohort study examines the incidence of sport-related and non–sport-related concussions among US undergraduate college students and varsity athletes stratified by sex and cause of concussion.

## Introduction

Concussion, a traumatically induced transient disturbance of brain function that involves a complex pathophysiological process,^1^ is of increasing concern among medical practitioners, public health specialists, researchers, sport organizations, and the public in general. To date, most concussion research has focused on sport-related concussion (SRC), although it is known that a substantial number of concussions occur outside of participating in sport. Focusing solely on SRC may result in an underestimation of the underlying incidence and prevalence of concussion in the general population and leads to the widely held perception that most concussions are sport related. An understudied population at risk for concussion is US undergraduate college students. While previous reports indicate that concussion incidence peaks between the ages of 9 and 22 years,^2^ there is a paucity of research focused on the older end of that range (ie, 18-22 years) that includes the traditional college student population. As such, this study attempts to characterize concussion incidence in an undergraduate student population throughout the academic year.

## Methods

This study was reviewed by the University of Colorado institutional review board and deemed exempt from approval and informed consent because the data were previously collected for clinical use and were deidentified prior to analysis. This study is reported following the Strengthening the Reporting of Observational Studies in Epidemiology (STROBE) reporting guideline.

### Study Population

Data were from a large, public university in the United States. They were derived from 2 sources: undergraduate students who sought medical care at the student health care center during the 2015 to 2016, 2016 to 2017, and 2017 to 2018 academic school years, and varsity student-athletes who received care through the university’s athletics department during the 2016 to 2017 and 2017 to 2018 academic school years.

### Concussion Diagnoses

Concussion diagnoses among the general undergraduate study population were made by a concussion team, including 3 medical practitioners with specialized training in concussion diagnosis and management. Undergraduate students presenting with symptoms consistent with concussion completed a health history questionnaire outlining details of the injury and prior concussion and were administered the Post-Concussion Symptom Scale.^3^ The student then met with a concussion team practitioner to complete a detailed concussion history and was evaluated using a series of standardized assessments focused on reported symptoms, neurological status, postural stability, and oculomotor and vestibular domains. After the evaluation, if appropriate, the diagnosis of concussion was made, and a treatment program was initiated. Concussion diagnoses for varsity student-athletes were made by a team of medical practitioners through the university’s sports medicine department. Varsity athletes presenting with symptoms consistent with concussion were evaluated using a series of standardized assessments focused on reported symptoms, neurological status, postural stability, and oculomotor and vestibular domains. Injury details for varsity athletes included whether the concussion was an SRC or non-SRC. Sport-related concussions for the general undergraduate population were defined as those occurring during organized, competitive, usually team-based competitions that included club and intramural sports but did not include participation in National Collegiate Athletic Association Division 1 athletics. Non–sport-related concussions included falls, hits to the head, motor vehicle crashes, and other recreational activities, such as bicycling and alpine skiing. Concussions that occurred during the months of May, June, and July as well as during holiday breaks were not included in the analysis.

### Statistical Analysis

We calculated 2 measures of concussion incidence: (1) academic-year annual incidence per 10 000 students, which included the general undergraduate population and varsity athletes, and (2) incidence per 1000 person-months to allow for month-to-month comparison, which included the general undergraduate population only. Incidence per 10 000 students was calculated by dividing the number of concussion diagnoses in an academic year by the total undergraduate enrollment for that same academic year and multiplying the result by 10 000. Monthly incidence rates were calculated by dividing the number of concussions in a given month by total person-months and is reported as the number of concussions per 1000 person-months. Person-months were calculated by multiplying the proportion of the months that students were on campus (excluding times such as holiday breaks and incomplete months) by the undergraduate student enrollment for each academic year. University undergraduate student enrollment information was extracted from the Office of Data Analytics for the 2015 to 2016, 2016 to 2017, and 2017 to 2018 academic years. We calculated 95% CIs using an exact method for Poisson counts. Information on which month the injury occurred for varsity athletes was not available.

Statistical analysis was performed using R statistical software version 3.6.1 (R Project for Statistical Computing). Data were analyzed from April to June 2019.

## Results

### General Undergraduate Population

A total of 1020 incident concussions from 954 undergraduate students were collected during the 2015 to 2016, 2016 to 2017, and 2017 to 2018 academic years. As a point of reference for the 954 undergraduate students diagnosed with concussion, the student health clinic treated 4864 students for nonconcussion musculoskeletal injuries and 16 029 students for other (nonconcussion, nonmusculoskeletal) conditions during the same period. Of 954 students diagnosed with at least 1 concussion, 502 (52.6%) were men and 452 (47.4%) were women. Racial/ethnic distribution included 750 white students (78.6%), 81 Hispanic students (8.5%), 59 Asian students (6.2%), 27 American Indian or Alaskan Native students (2.8%), 18 black students (1.9%), and 19 students (2.0%) who reported their race/ethnicity as other or whose race/ethnicity was not reported, which approximated the racial/ethnic distribution of the university student body as a whole for those academic years. In all, 393 students (41.2%) reported no prior concussions, 395 students (41.3%) reported 1 to 3 previous concussions, and 47 students (4.9%) reported 4 or more prior concussions (119 students [12.4%] did not report their prior concussions). Causes of concussion included falls (385 concussions [37.7%]), sport related (363 concussions [35.6%]), hit to the head (87 concussions [8.5%]), motor vehicle crashes (66 concussions [6.5%]), other (108 concussions [10.6%]), and missing or unknown reason (11 concussions [1.1%]). Descriptive statistics of the general undergraduate study sample are displayed in Table 1.

**Table 1.** Characteristics of Concussion Diagnoses Among Undergraduate Students

Characteristic	Students, No. (%)	
	General Undergraduate Population^a^	Varsity Athletes^b^
Concussions diagnosed	1020	80
Sex		
Women	452 (47.4)	54 (67.5)
Men	502 (52.6)	26 (32.5)
Race/ethnicity		
White	750 (78.6)	NA
Black	18 (1.9)	NA
Hispanic	81 (8.5)	NA
Asian	59 (6.2)	NA
American Indian or Alaska Native	27 (2.8)	NA
Other or unknown	19 (2.0)	NA
Prior concussions		
0	393 (41.2)	NA
1-3	395 (41.4)	NA
≥4	47 (4.9)	NA
Unknown or missing	119 (12.4)	NA
Cause		
Non–sport-related	646 (63.3)	14 (17.5)
Fall	385 (37.8)	NA
Hit to the head	87 (8.5)	NA
Motor vehicle crash	66 (6.5)	NA
Other	108 (10.6)	NA
Sport-related	363 (35.6)	66 (82.5)
Unknown or missing	11 (1.1)	0

Abbreviation: NA, not available.

^a^ Includes academic years 2015 to 2016, 2016 to 2017, and 2017 to 2018.

^b^ Includes academic years 2016 to 2017 and 2017 to 2018.

### Varsity Athletes

In the 2016 to 2017 academic year, there were 11 concussions (9 SRCs, 2 non-SRCs) among 238 men athletes and 32 concussions (28 SRCs, 4 non-SRCs) among 221 women athletes. In the 2017 to 2018 academic year, there were 15 concussions (10 SRCs, 5 non-SRCs) among 229 men athletes and 22 concussions (19 SRCs, 3 non-SRCs) among 219 women athletes. Information on the varsity athlete population is displayed in Table 1.

### Concussion Incidence

Concussion incidence by academic year is reported in Table 2 and further stratified by sex in Table 3. Overall academic year incidence during the course of the study was 121.5 (95% CI, 114.3-129.1) concussions per 10 000 students among the general undergraduate population only and 132.4 (95% CI, 123.2-142.0) concussions per 10 000 students when including varsity athletes. Among the general undergraduate population, men sustained concussions at a rate of 115.7 (95% CI, 106.4-125.8) concussions per 10 000 students, and women sustained concussions at a rate of 128.8 (95% CI, 117.8-140.7) concussions per 10 000 students. When incorporating concussion from varsity athletes, men sustained concussions at a rate of 126.1 (95% CI, 114.1-139.0) concussions per 10 000 students and women sustained concussions at a rate of 140.0 (95% CI, 126.2-155.3) concussions per 10 000 students for the 2016 to 2017 and 2017 to 2018 academic years.

**Table 2.** Concussion Incidence by Academic Year

Academic Year	Study Population	Concussions, No.	No. of Concussions per 10 000 Students (95% CI)
2015-2016	General undergraduate	347	128.5 (115.7-142.6)
	General undergraduate and varsity athletes	NA	NA
2016-2017	General undergraduate	343	123.2 (110.9-136.8)
	General undergraduate and varsity athletes	386	138.6 (125.5-153.1)
2017-2018	General undergraduate	330	113.4 (101.9-126.3)
	General undergraduate and varsity athletes	367	126.2 (114.0-139.7)
Overall	General undergraduate	1020	121.5 (114.3-129.1)
	General undergraduate and varsity athletes^a^	753	132.4 (123.2-142.0)

Abbreviation: NA, not available.

^a^ For academic years 2016 to 2017 and 2017 to 2018.

**Table 3.** Concussion Incidence for Men and Women Stratified by Academic Year

Academic Year	Study Population	Men		Women	
		Concussions, No.	No. of Concussions per 10 000 Students (95% CI)	Concussions, No.	No. of Concussions per 10 000 Students (95% CI)
2015-2016	General undergraduate	166	111.1 (95.5-129.2)	181	150.0 (129.8-173.3)
	General undergraduate and varsity athletes	NA	NA	NA	NA
2016-2017	General undergraduate	194	125.2 (108.9-144.0)	149	120.6 (102.8-141.5)
	General undergraduate and varsity athletes	205	132.3 (115.5-151.6)	181	146.5 (126.8-169.3)
2017-2018	General undergraduate	180	110.9 (95.9-128.2)	150	116.7 (99.5-136.8)
	General undergraduate and varsity athletes	195	120.1 (104.5-138.1)	172	133.8 (115.3-155.2)
Overall	General undergraduate	540	115.7 (106.4-125.8)	480	128.76 (117.8-140.7)
	General undergraduate and varsity athletes^a^	400	126.1 (114.4-139.0)	353	140.0 (126.2-155.3)

Abbreviation: NA, not available.

^a^ For academic years 2016 to 2017 and 2017 to 2018 only.

Occurrence of non-SRC exceeded SRC during all academic years under study. There were 363 SRCs and 646 non-SRCs among the general undergraduate population across the 3 academic years. When data from varsity athletes were included for academic years 2016 to 2017 and 2017 to 2018, there were 293 SRCs and 460 non-SRCs. Across the 3 academic years excluding varsity athletes, the incidence of SRC was 43.2 (95% CI, 39.0-47.9) concussions per 10 000 students. When including the varsity athletes, SRC incidence was 51.5 (95% CI, 49.5-57.7) concussions per 10 000 students for academic years 2016 to 2017 and 2017 to 2018. For non-SRC, the incidence for academic years 2016 to 2017 and 2017 to 2018 was 77.0 (95% CI, 71.3-83.1) concussions per 10 000 excluding varsity athletes and 81.0 (95% CI, 73.9-88.7) concussions per 10 000 students including varsity athletes.

Incidence rates by month and reported cause are presented in the Figure for the general undergraduate population. Overall incidence (4.5 [95% CI, 3.5-5.6] concussions per 1000 person-months) and non-SRC incidence (1.8 [95% CI, 1.2-2.6] concussions per 1000 person-months) were highest in August. Incidence of SRC was relatively consistent throughout the academic year and lower than non-SRC throughout all months of observation.

**Figure.**
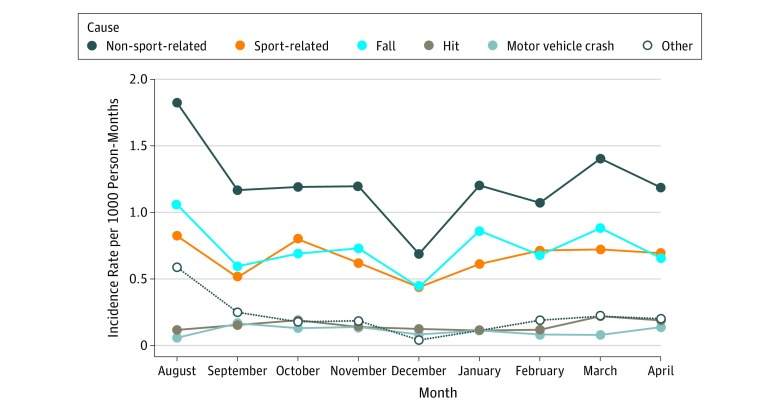
Concussion Incidence by Cause During the Academic Year for the General Undergraduate Population Non–sport-related concussions include concussions caused by falls, hits, motor vehicle crashes, and other causes.

## Discussion

Concussions are common among the collegiate student population as measured at a large, public US university. Incidence in this sample—121.5 concussions per 10 000 students without varsity athletes and 132.4 concussions per 10 000 students with varsity athletes—was more than 2-fold that reported by the World Health Organization^4^ in the general population (600 concussions per 100 000 people) in 2004. Of note, the World Health Organization incidence measure is based upon a full 12-month year, whereas we report in this study the concussion incidence from a 9-month academic year. Concussion incidence for this study population is also higher than that reported for people aged 9 to 22 years from the US Centers for Disease Control and Prevention (from 576.9 concussions per 100 000 people in 2001 to 2002 to 981.9 concussions per 100 000 people in 2009 to 2010).^5,6^ These studies used emergency department visits as their data set, which could play a role in lower incidence rates as the threshold to present to an emergency department is presumably higher. Our findings suggest that collegiate students, including the general population and varsity athletes, may be at an increased risk of concussion compared with the general population.

We found that concussions were common among men and women in this population. In fact, women had a slightly higher overall incidence of concussion compared with men during 2 of the 3 academic years under study for the general undergraduate population and women had a higher rate of concussions as varsity athletes compared with men. Consistent with this finding, a recent study in people aged 5 to 18 years reported a 6.3-fold increase in concussions among girls and women from 2003 to 2013 compared with a 3.6-fold increase in boys and men.^7^ Both anatomical and symptom reporting differences have been hypothesized as reasons for increased concussion rates among women.

Additionally, we characterized the variability in concussion incidence by cause throughout the academic year for the general undergraduate population. The incidence of SRC remained stable throughout the academic year, with a notable reduction during December corresponding to the end of the fall academic semester. The reduced incidence in December may be because students engaged in fewer activities owing to academic demands (eg, final examinations, projects, essays) at the end of the semester. In contrast, non-SRC incidence varied substantially throughout the academic year.

### Limitations

This study has limitations. Not all students will seek care for a concussion, and not all who do will seek care at the student health care center. Furthermore, it is likely that not all varsity athletes will report their concussion symptoms to medical practitioners. Therefore, it is likely that the reported incidence rate is an underestimation of the true incidence rate. It is also unclear if the general undergraduate population seeking care at the student health care center is an accurate representation of the university’s student body and, beyond that, whether the study university’s student body is representative of other US collegiate populations.

## Conclusions

This cohort study found that concussions among US undergraduate students were common and occurred equivalently between men and women proportionate to university sex distribution. Incidence of concussion in undergraduate students was higher than that reported in other studies examining this age range and, importantly, were more commonly found to be caused by non–sport-related activity compared with sport-related activity. Our findings suggest that concussion among US undergraduate students is a significant health care burden for student health care centers.
